# Pathology and causes of death in captive meerkats (*Suricata suricatta*)

**DOI:** 10.1080/01652176.2023.2211120

**Published:** 2023-05-15

**Authors:** Bernat Martí-García, Simon L Priestnall, Alejandro Suárez-Bonnet

**Affiliations:** Pathobiology & Population Sciences, Royal Veterinary College, Hertfordshire, UK

**Keywords:** Meerkat, *Suricata suricatta*, pathology, disease, atherosclerosis

## Abstract

**Background:**

Meerkats (*Suricata suricatta*) are endemic carnivores of southern Africa and, although currently listed as ‘least concern’ by the International Union for Conservation of Nature (IUCN) red list, there is evidence of a significant decrease in wild populations mainly attributed to effects of climate change. Little is known about diseases associated with mortality in captive meerkats.

**Aim:**

To characterise macroscopic and microscopic lesions that accounted for the death or euthanasia in a series of captive meerkats.

**Material and methods:**

Eight captive meerkats submitted for post-mortem examination between 2018 and 2022.

**Results:**

Three animals died unexpectedly without clinical signs, 2 exhibited neurological signs, 2 collapsed after con-specific fighting and 1 showed gastrointestinal signs. Common pathological findings of this study that may be related to the death of captive meerkats included foreign bodies (trichobezoars or plastic materials) within the alimentary tract, traumatic penetrating injuries or starvation associated with abnormal social behaviours (bullying and con-specific attacks), verminous pneumonia and systemic atherosclerosis. Common incidental findings included pulmonary edema and congestion, cholesterol granulomas, pulmonary adenomas and vertebral spondylosis.

**Conclusions:**

Non-infectious diseases outreach infectious diseases as causes of mortality in captive meerkats including, foreign bodies within the alimentary tract, con-specific attacks and systemic atherosclerosis, which is described for the first time. These data should raise concern about appropriate husbandry (e.g. environmental enrichment, cleaning of facilities and diet formulation) by zookeepers and emphasise the need for further study of meerkat mortality in both captive and wild populations.

## 1. Introduction

Meerkats (*Suricata Suricatta*) are small diurnal, and markedly gregarious animals belonging to the order Carnivora and family Herpestidae. They inhabit the plains of Southern Africa, including savannas, grasslands and low hills and their diet is highly diverse: chiefly insects, hunted vertebrates (lizards and birds) and gathered fruit (Brox et al. 2021). Meerkats are a highly social species and con-specific interactions between groups and individuals are common behaviours, especially by means of communication or vocalisation (e.g. purring sounds and shrill calls) and cooperation skills used for hunting, breeding, guarding and, occasionally, for travelling long distances (Kranstauber et al. 2020; Huels and Stoeger 2022). One of the main causes of mortality in free-ranging meerkats is predation by birds and larger terrestrial carnivores.

Meerkats are currently listed as ‘least concern’ by the International Union for Conservation of Nature (IUCN) red list. However, Paniw et al. recently indicated that climate change acts as a catalyst for the extinction of this species by significantly reducing the adult population in the pre-breeding season and the fecundity rates during the breeding season (Paniw et al. 2019). Understanding the pathology and biology of meerkats is pivotal to support the conservation of this species.

Diseases and causes of mortality in captive meerkats are sporadically reported and are even more scarcely described in free-ranging meerkats (Drewe et al. 2009). Nonetheless, diseases in this species can be broadly classified as infectious and non-infectious. Regarding infectious diseases, there is considerable overlap in disease susceptibility between Herpestidae and the well-studied Felidae and Canidae families, such as toxoplasmosis and distemper (Coke et al. 2005; Basso et al. 2009). Susceptibility to and potential reservoir status for zoonotic diseases such as avian influenza virus and coronaviruses (e.g. SARS-Coronavirus 1) have recently been reviewed (Wicker et al. 2017). Other important infectious diseases of zoonotic relevance in meerkats are tuberculosis (Drewe et al. 2009), melioidosis (Rachlin et al. 2019), Japanese encephalitis (Piewbang et al. 2022), rabies (Koeppel et al. 2022) and yersiniosis (Nakamura et al. 2015). The existence of shared diseases between meerkats and other species raises serious concerns about their role as potential reservoirs and spreaders of life-threatening pathogens to humans. Non-infectious diseases, namely neoplasia, are rarely reported and consist of individual case reports in captive meerkats. Neoplasms in this species are reported as incidental findings with no previous or little clinical signs, but also associated with systemic disease. Spontaneous neoplasms described in meerkats include liposarcoma (Aihara and Une 2009), pelvic nephroblastoma, mandibular carcinoma (Dadone et al. 2014) and hepatocellular carcinoma and cholangiocarcinoma and (Boonsri et al. 2013; Marrow et al. 2014). Other non-infectious causes of death or euthanasia of captive meerkats include traumatic events due to con-specific attacks, especially during feeding times (Tomczyk and Zieliński 2021) and heart valvular dysplasia (Chai et al. 2004; Cham et al. 2022).

To the authors’ knowledge, overall descriptive pathology and causes of mortality in meerkats are lacking, with only case reports describing specific diseases or conditions in meerkats having been published to date. Therefore, the objective of this study is to review the pathology and the most common causes of mortality in captive meerkats from two local zoos in the UK. We also aimed to highlight the importance of keeping pathological records from deceased captive animals by means of thorough post-mortem (PM) examinations carried out by board-certified pathologists on a regular basis and the use of ancillary tests, particularly in cases of sudden or unexpected death.

## 2. Materials and methods

Gross and histologic data of 8 captive meerkats (*Suricata suricatta*) that were submitted from 2018 to 2022 were collected retrospectively from the archives of the Diagnostic Laboratories of the Royal Veterinary College (RVC), UK. The carcasses were submitted from two local zoos for full pathological examination. Four animals died naturally, and 4 animals were humanely euthanised due to poor clinical prognosis and welfare issues. There were 7 males and 1 female. Ages ranged from 2 to 10 years (mean = 6.3 yr and median = 7yr). Their diet consisted of dry feline commercial diet, fruits and vegetables.

Complete necropsy procedures were conducted on each case as per standard protocol followed in Diagnostic Laboratories of the RVC. Body condition was graded as follows: 1) cachexia (moderate to severe muscle mass atrophy and serous atrophy of adipose stores), 2) poor condition (imperceptible to mild muscle mass atrophy with depletion of peri-visceral adipose stores, but without evidence of serous atrophy) or 3) normal to good condition (no depletion of musculature, presence of subcutaneous and perivisceral adipose stores).

Tissue sections for histopathological evaluation were fixed in 10% neutral buffered formalin, routinely processed, sectioned at 4 µm, stained with haematoxylin and eosin and examined with a light microscope. In selected cases (cases 4-7) additional special stains, including Gram, Toluidine Blue, Luxol Fast Blue and Masson’s Trichrome were used. Immunolabelling was performed on paraffin-embedded sections of lung tissue from one animal (case 3) using anti-TTF-1, multicytokeratin (MCK), vimentin, Ki67, EGF-R, COX-2, PD1, PD-L1, SOX-9, antibodies (Table 1). Qualitative PCRs for canine distemper virus (CDV) (NP gene) and nematodes (18S rRNA and ITS2 regions) were performed in one animal (case 2).

**Table 1.** Summary of immunohistochemical methodology.

Antibody	Source	Host	Type	Clone	Antigen Retrieval	Dilution
AE1/AE3	Dako	Mouse	Monoclonal	AE1 y AE3	Citrate buffer	1:100
Vimentin	Dako	Mouse	Monoclonal	V9	Citrate buffer	1:100
TTF1	Abcam	Rabbit	Monoclonal	SP141	Tris-EDTA buffer	1:100
COX-2	ThermoFisher	Mouse	Monoclonal	SP21	Citrate buffer	1:50
EGFR	ThermoFisher	Mouse	Monoclonal	MA5-13070	Citrate buffer	1:100
Ki67	Dako	Mouse	Monoclonal	MIB-1	Citrate buffer	1:200
PD1	Abcam	Rabbit	Monoclonal	EPR20665	Tris-EDTA buffer	1:500
PD-L1	ThermoFisher	Rabbit	Monoclonal	JJ08-95	Tris-EDTA buffer	1:100
SOX-9	Sigma	Rabbit	Polyclonal	HPA001758	Citrate buffer	1:200

## 3. Results

Table 2 summarizes the main clinical signs, pathological findings and causes of death for the 8 cases.

**Table 2.** Summary of clinical signs, pathological findings and causes of death for the 8 meerkats in captivity.

Case	Sex	Age (years)	Body weight (kg)/Body condition	Clinical history	Macroscopic findings	Histopathological findings	Cause of death/euthanasia
1	Male	6	1.005/Normal-Good	Sudden death	Plastic duodenal foreign body, hemoabdomen, peritonitis	Fibrinonecrotizing and haemorrhagic enteritis	Natural death Intestinal perforation, peritonitis and septic shock
2	Male	4	0.873/Normal-Good	Seizures	Lung consolidation, hepatopathy, hemoabdomen	Verminous granulomatous pneumonia, lymphoplasmacytic meningitis and myocarditis	Euthanasia Multifactorial: verminous pneumonia, meningitis and myocarditis.
3	Male	10	1.050/Normal-Good	Sudden death	Subcutaneous edema, systemic yellow nodules, hepatopathy, hydrothorax, ascites, hydrocephalus, myocardial necrosis, splenic nodular hyperplasia, pulmonary white nodules	Systemic cholesterol granulomas, hepatic lipidosis, myocardial necrosis with auricular thrombosis, splenic lymphoid hyperplasia, bronchoalveolar adenomas, glomerulosclerosis	Natural death Systemic atherosclerosis
4	Female	8	0.690/Poor	Head tilt, ataxia, depression, paraparesis	Poor body condition, dental disease, spondylosis, liver and lung congestion, pulmonary white nodule	Multifocal mineralisation and fibrosis of the stomach (tunica muscularis), bronchoalveolar adenoma	Euthanasia on welfare grounds
5	Male	Unknown	0.835/Normal-Good	Sudden death	Penetrating cutaneous wounds, subcutaneous haematoma, haemothorax, pulmonary rupture	Pulmonary haemorrhages with atelectasis, pleuritis, hepatic centrilobular necrosis	Natural death Hypovolemic shock with respiratory insufficiency
6	Male	Unknown	0.770/Normal-Good	Collapsed after con-specific attack	Penetrated cutaneous wounds, chronic dermatitis, gastric trichobezoar	Suppurative chronic dermatitis with intralesional bacteria, hepatic lipidosis; pulmonary congestion, subpleural emphysema, mineralization of choroid plexuses	Euthanasia on welfare grounds
7	Male	2	0.717/Poor	Collapsed after con-specific attack	Poor body condition, sunken eyes, muscle atrophy, gastric trichophytobezoar, empty intestinal tract	Pulmonary atelectasis, hepatic lipidosis	Natural death Starvation
8	Male	8	0.930/Normal-Good	Vomiting, lethargy, cervical mass	Plastic esophageal foreign body, pulmonary yellow nodule, hepatopathy	Focal esophageal erosion, focal pulmonary cholesterol granuloma	Euthanasia on welfare grounds

### 3.1. External examination and integumentary system

**Figure 1.**
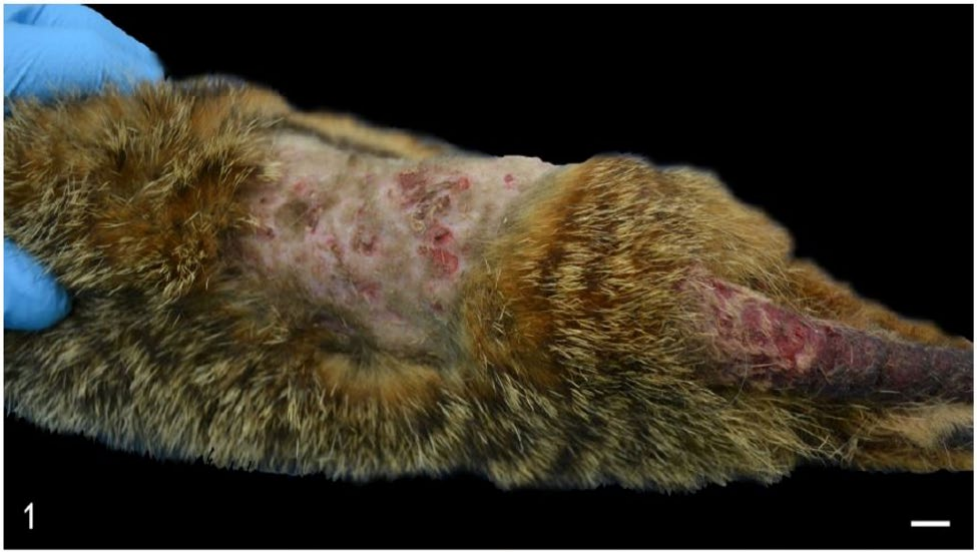
Case 6. The dorsum and tail exhibit locally extensive area of chronic dermatitis with alopecia, crusting, hyperkeratosis and multifocal erosions and ulcers. Bar = 1 cm.

Two animals (cases 4 and 7) were classified as having a poor body condition while the rest were in normal to good body condition. Two animals (cases 4 and 8) showed dental disease and one animal (case 3) exhibited subcutaneous oedema of the ventral abdomen. Two animals (cases 1 and 4) had spondylosis. Cutaneous and subcutaneous traumatic lesions, consisting of skin tearing with subcutaneous and muscular haemorrhages and/or tearing was observed in cases 5 and 6. Skin lesions in case 6 were markedly chronic with crusting and thickening of the lumbar skin (Figure 1). Histopathology of skin from case 6 revealed chronic dermatitis with abundant Gram-positive coccoid bacteria and moderate numbers of dermal perivascular mast cells in case 6.

### 3.2. Body cavities

**Figure 2.**
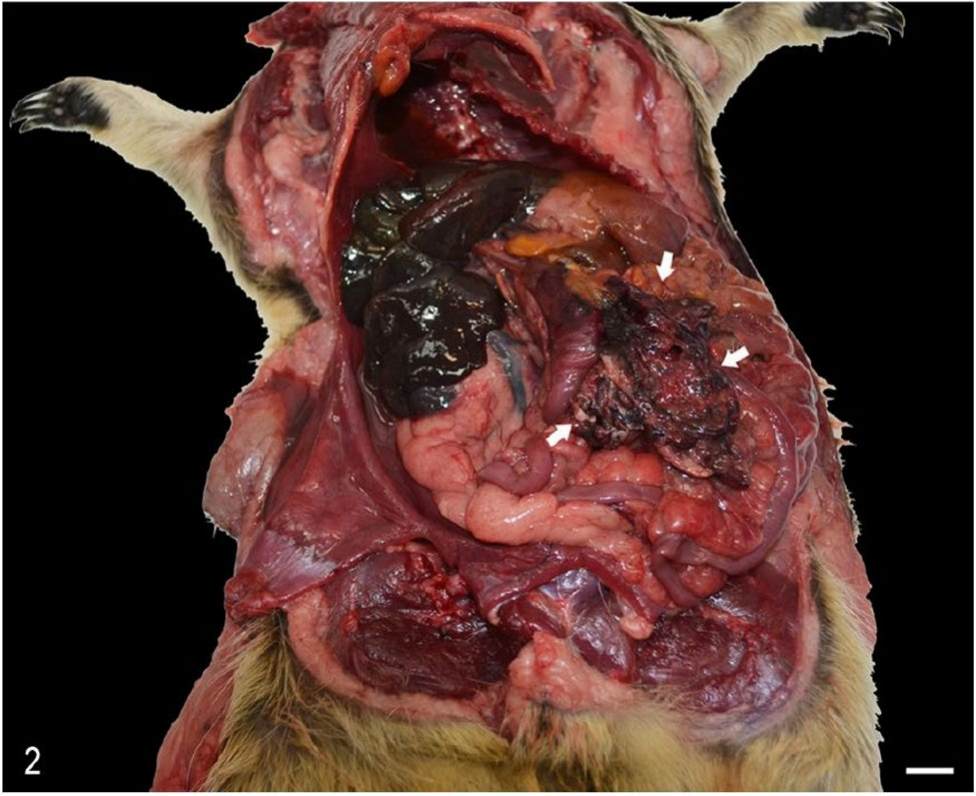
Case 1. Focally extensive peritoneal hemorrhage (arrows) as a result of a foreign body-induced duodenal perforation. Bar = 1 cm.

Straw-coloured, clear free fluid was found in the abdominal cavity (ascites) of 1 animal (case 3) and in the thoracic cavity (hydrothorax) of 2 animals (cases 3 and 4). Haemothorax was found in 1 animal (case 5) whereas hemoabdomen was identified in 2 animals (cases 1 and 2) (Figure 2).

### 3.3. Digestive system

**Figure 3.**
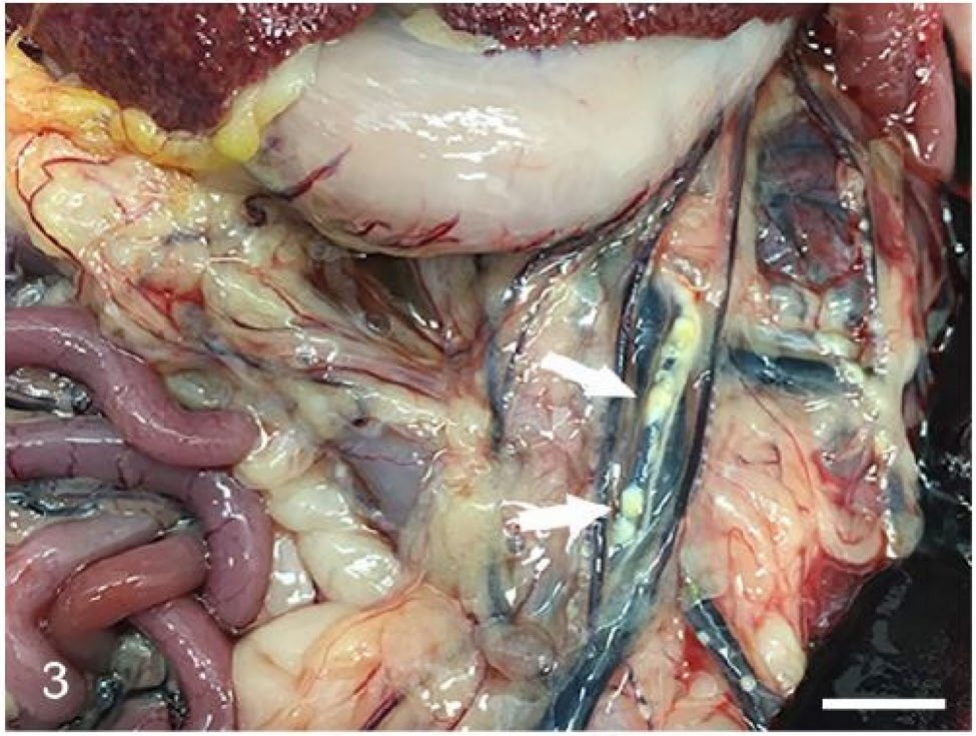
Case 3. The short gastric arteries are expanded by multifocal pale-yellow (atheromatous) plaques (arrows).

One animal (case 3) showed multifocal yellow, firm plaques expanding the wall of the small gastric arteries (Figure 3) which microscopically were mineralized atheromatous plaques. Gastrointestinal content was variable, from absent (case 7) to small amounts (cases 1-6 and 8). Four animals (cases 1, 6, 7 and 8) showed foreign bodies in the digestive system that corresponded with gastric tricho/phytobezoars (in cases 6 and 7) or with plastic foreign bodies measuring 2 × 1 cm and 3 × 1.5 cm located in the duodenum and esophagus, respectively (cases 1 and 8). The former caused a local perforation, hemoabdomen and a focally extensive peritonitis. Histopathologically, the duodenum showed transmural necrosis and suppurative inflammation that extended into the peritoneum. The esophageal foreign body displaced and compressed the larynx and the proximal trachea. Microscopically, the esophageal mucosa was eroded.

### 3.4. Liver and spleen

Hepatopathy with or without hepatomegaly was grossly detected in 3 animals (cases 2, 3 and 8) whereas hepatic congestion was observed in 4 animals (cases 1,3, 4 and 8). Microscopically hepatopathy consisted of lipidic degeneration. Splenomegaly was observed in case 3 and histopathologically consisted of lymphoid follicular (white pulp) hyperplasia.

### 3.5. Cardiovascular system

**Figure 4.**
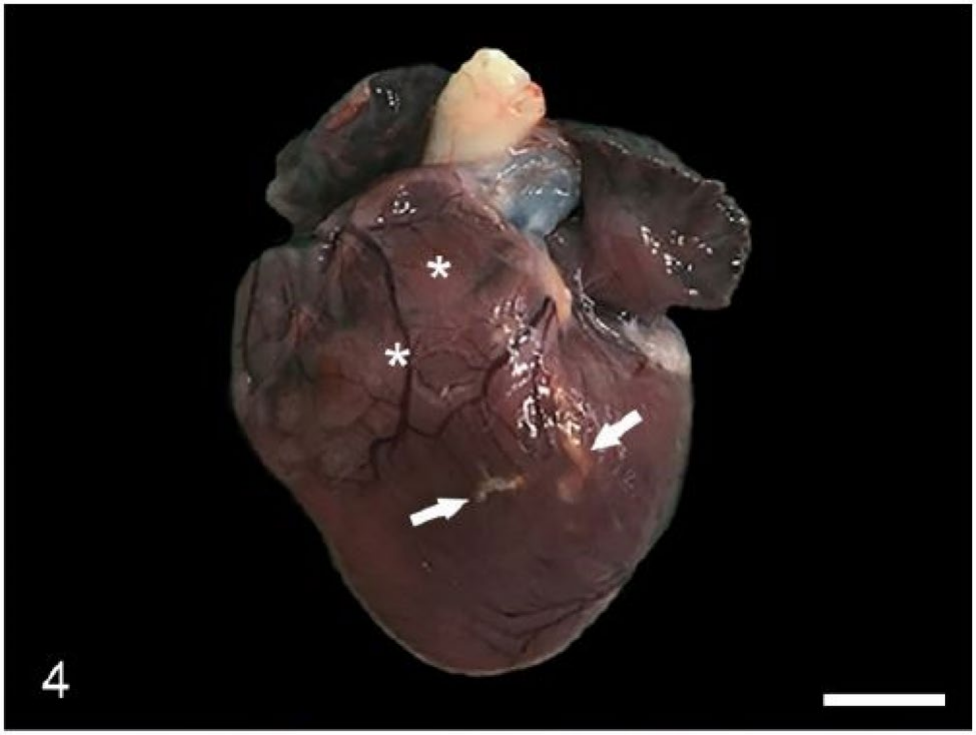
Case 3. The heart exhibits tortuous, yellow, stripes (arrows) that correspond with atheromatous branches of the coronary arteries, and pale areas of myocardium that correspond with areas of degeneration (asterisks).

**Figure 5.**
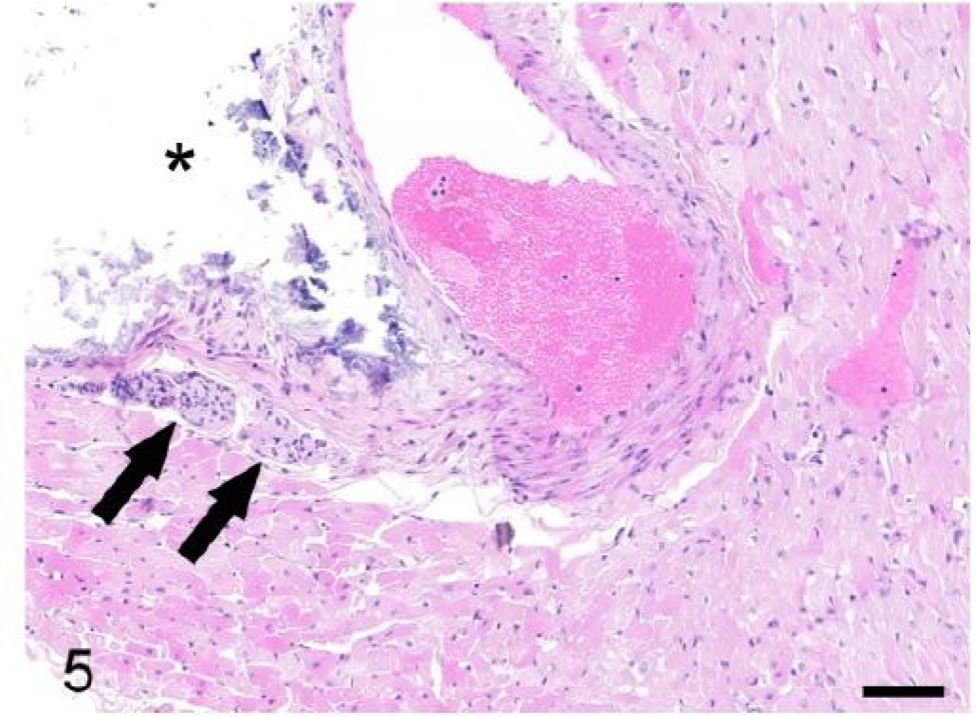
Case 3. The tunica media of a coronary artery is expanded by a mineralized atheromatous plaque (asterisk). The atheromatous plaque compresses a segment of the bundle of His (arrows). Bar = 50 µm.

**Figure 6.**
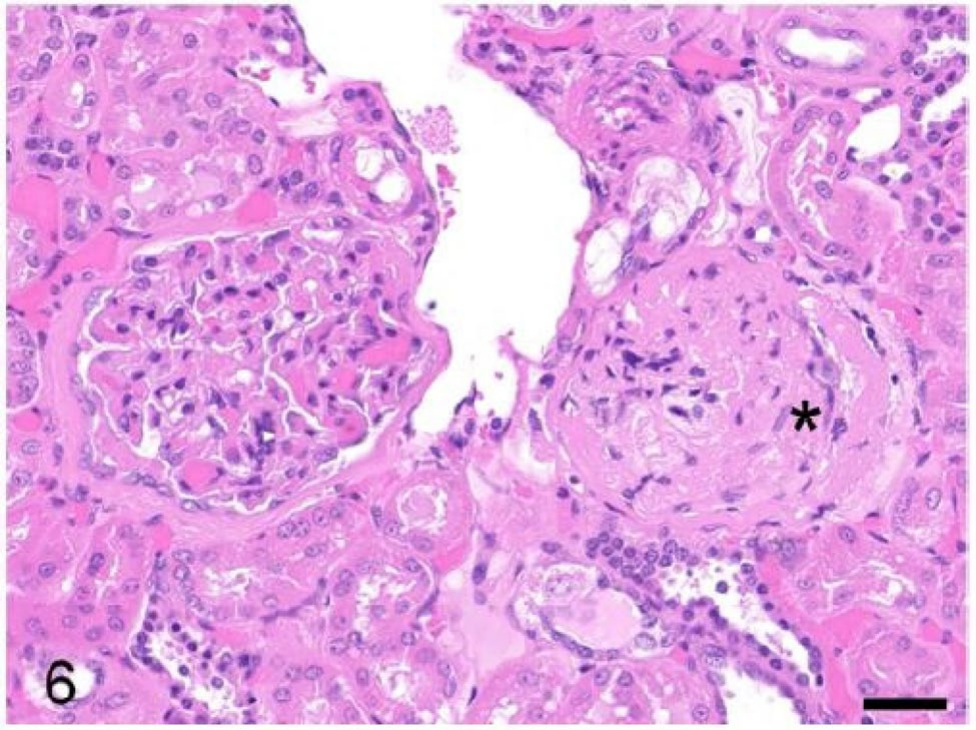
Case 3. Glomerulosclerosis. Glomerular capillaries are markedly thickened and degenerated. The Bowman’s capsule is also markedly thickened (asterisk). Bar = 20 µm.

**Figure 7.**
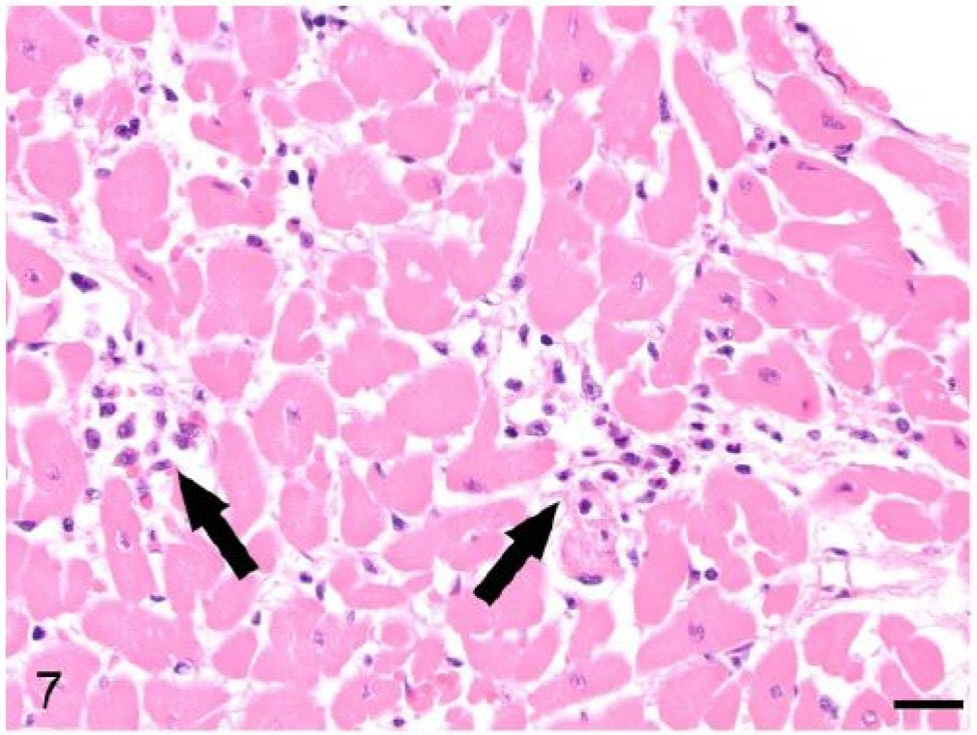
Case 2. The myocardium is infiltrated by lymphocytes, plasma cells and neutrophils (arrows). Bar = 20 µm.

The heart in case 3 exhibited tortuous, yellow, stripes involving the coronary arteries with pale discolouration of the myocardium (Figure 4). Microscopically these areas were atheromatous plaques involving the tunica media of the coronary arteries (Figure 5). Atheromatous plaques had a large, mineralized core surrounded by abundant clear acicular cholesterol crystals admixed with macrophages, lymphocytes and plasma cells. The myocardium exhibited multifocal degeneration and early necrosis, with hypereosinophilic or pale and fragmented cardiomyocytes. Case 3 also exhibited auricular thrombosis and evidence of vascular changes within the kidney consistent with glomerulosclerosis (Figure 6). Microscopic examination of the heart of case 2 revealed mild, multifocal neutrophilic and lymphoplasmacytic myocardial infiltrates (Figure 7) and segmental fibrinoid necrosis of the coronary vessels in one animal.

### 3.6. Nervous system

**Figure 8.**
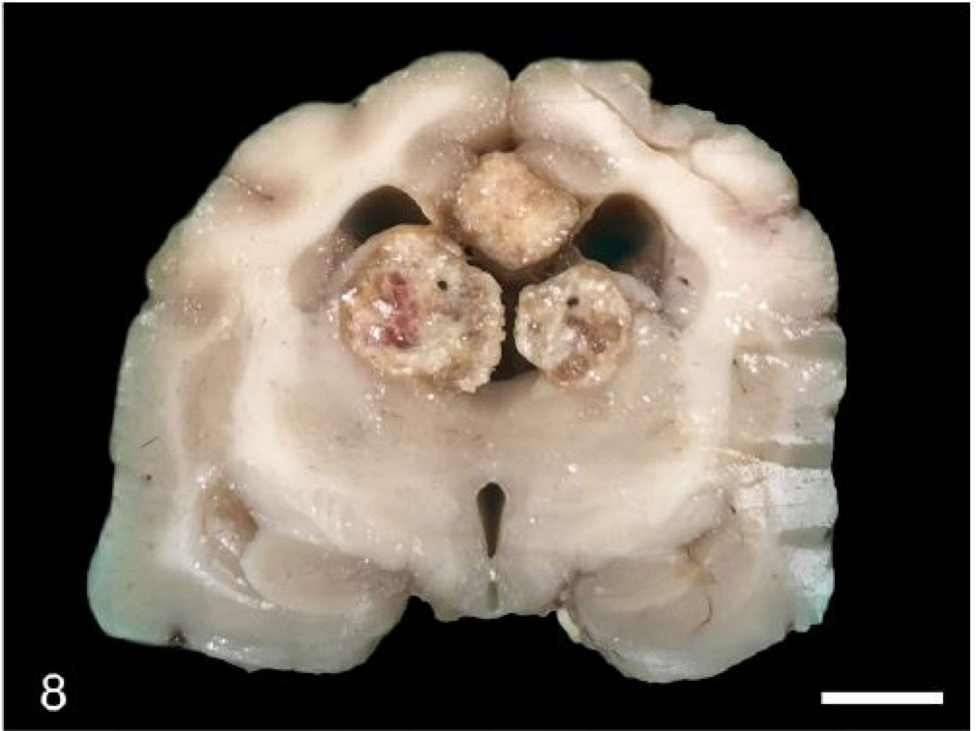
Case 3. Cholesterol granulomas expand the choroid plexus and compress the lateral ventricles and surrounding parenchyma. The third ventricle is dilated. Bar = 3 mm.

**Figure 9.**
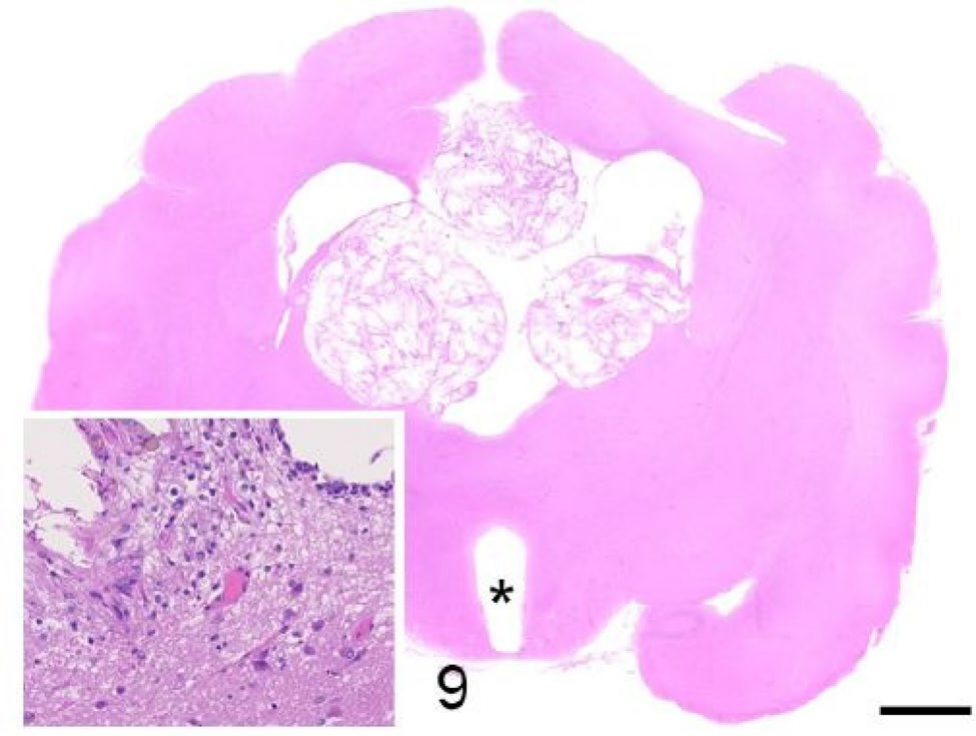
Case 3. A multinodular cholesterol granuloma compresses the lateral ventricles. The third ventricle is markedly dilated (asterisk). *Inset*: There is, hypercellularity, rarefaction and vacuolation of the neuropil (degeneration) and rare hemosiderin-laden macrophages (chronic haemorrhage). Bar = 3 mm.

Gross lesions were only observed in the brain in case 3, which showed bilateral marked hydrocephalus and a multinodular, yellow-tan, gritty and friable intraventricular mass measuring 1 cm in diameter (Figure 8). Microscopically, it consisted of choroid plexus cholesterol granulomas that compressed the surrounding neuropil (Figure 9). Multifocal dystrophic mineralisation of the choroid plexus was observed in one animal (case 6). Microscopic examination of the CNS of one animal (case 2) revealed mild lymphoplasmacytic meningitis. A qualitative PCR for CDV in this case yielded a negative result. The Luxol Fast Blue stain did not reveal evidence of demyelination in any case.

### 3.7. Respiratory system

**Figure 10.**
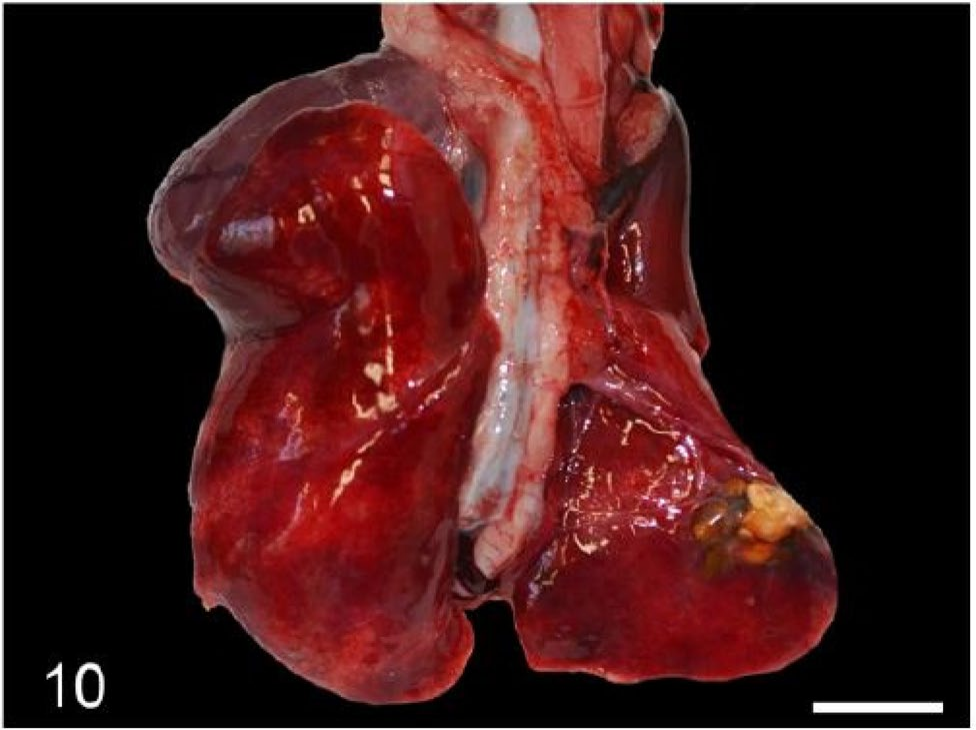
Case 8. Focal pulmonary cholesterol granuloma. Bars = 1 cm.

**Figure 11.**
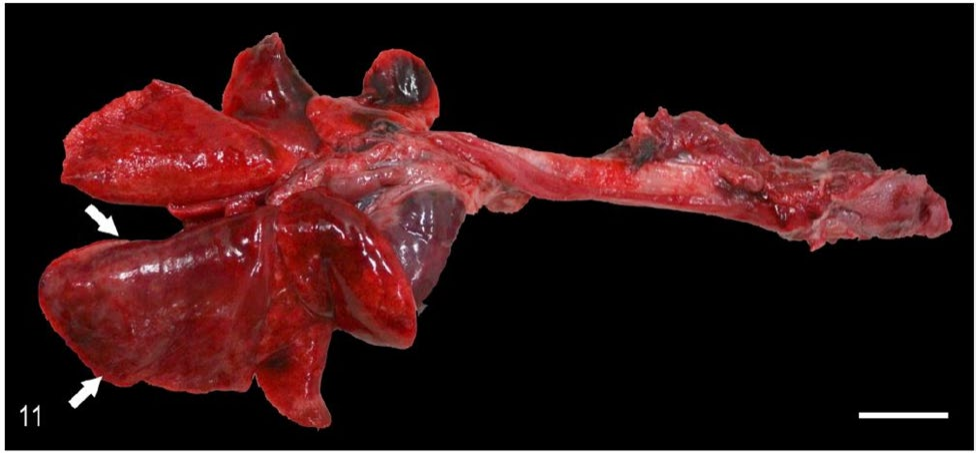
Case 2. Granulomatous pneumonia. The right caudal lung lobe is diffusely dark red, firm and enlarged (arrows). Bar = 1 cm.

**Figure 12.**
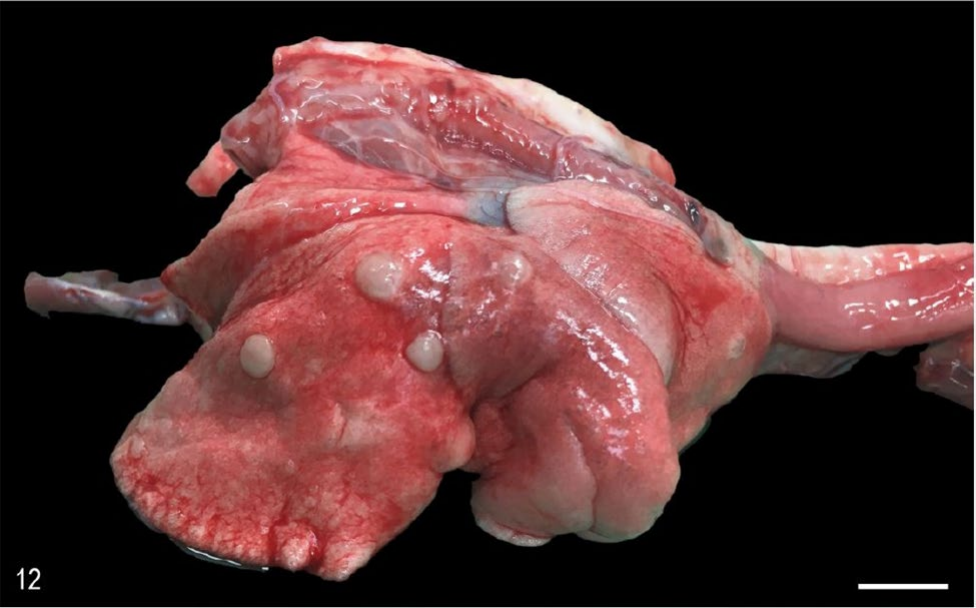
Case 3. Pulmonary adenomas. The right lung exhibits multifocal, white, firm, round nodules that rise the pleura. Bar = 1 cm.

**Figure 13.**
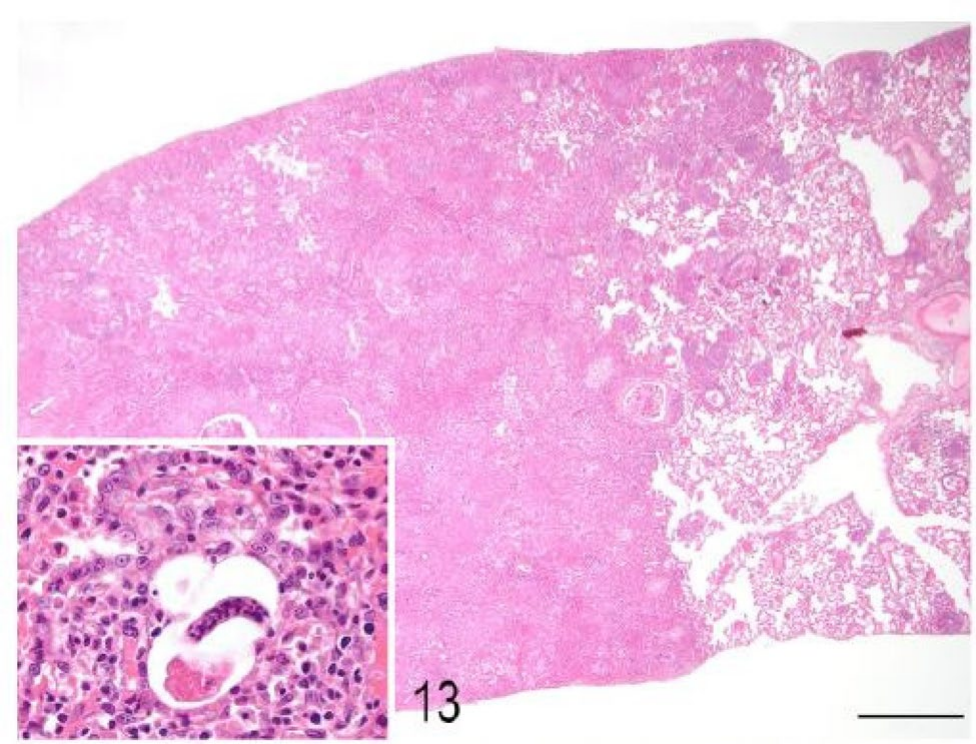
Case 2. Granulomatous pneumonia. The lung is locally extensively expanded by a pyogranulomatous inflammatory exudate (consolidation). *Inset*: A nematode larvae is surrounded by macrophages, eosinophils, lymphocytes, and plasma cells. There is marked pneumocyte type II hypertrophy. Bar = 1 mm.

**Figure 14.**
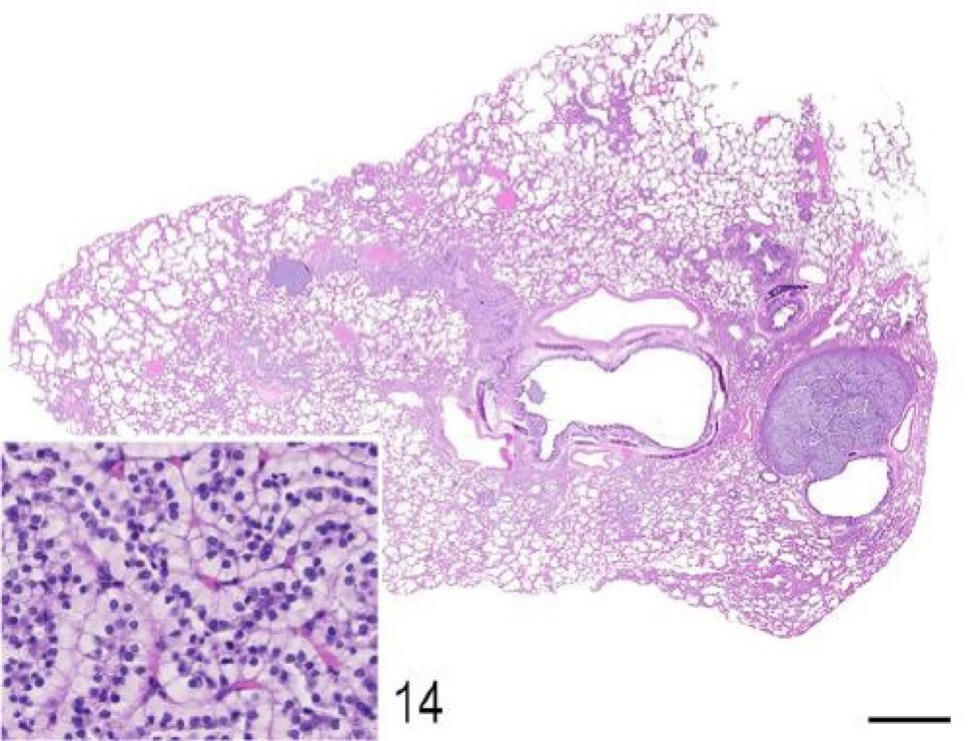
Case 3. Pulmonary adenomas. The lung parenchyma is expanded by variably sized well-demarcated epithelial neoplasms that compress the surrounding alveolar spaces. *Inset*: The neoplasms consist of clear cuboidal cells with defined margins and a single polarized nucleus. Cellular atypia is minimal. Bar = 1 mm.

**Figure 15.**
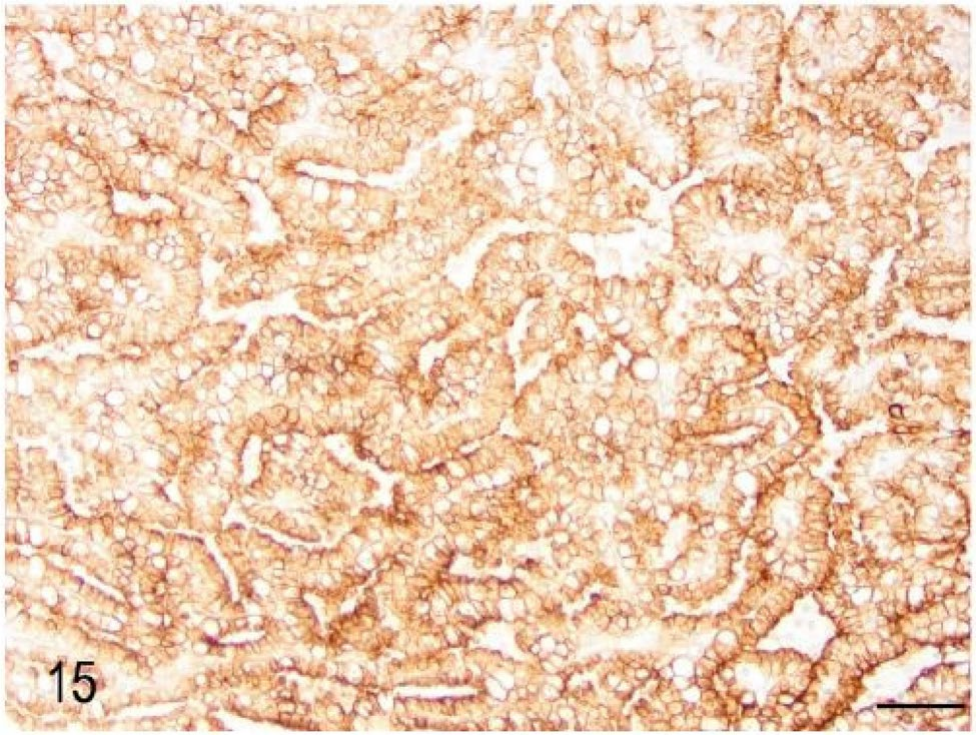
Case 3. The pulmonary adenoma was diffusely positive for multicytokeratin immunohistochemistry. Bar = 50 µm.

**Figure 16.**
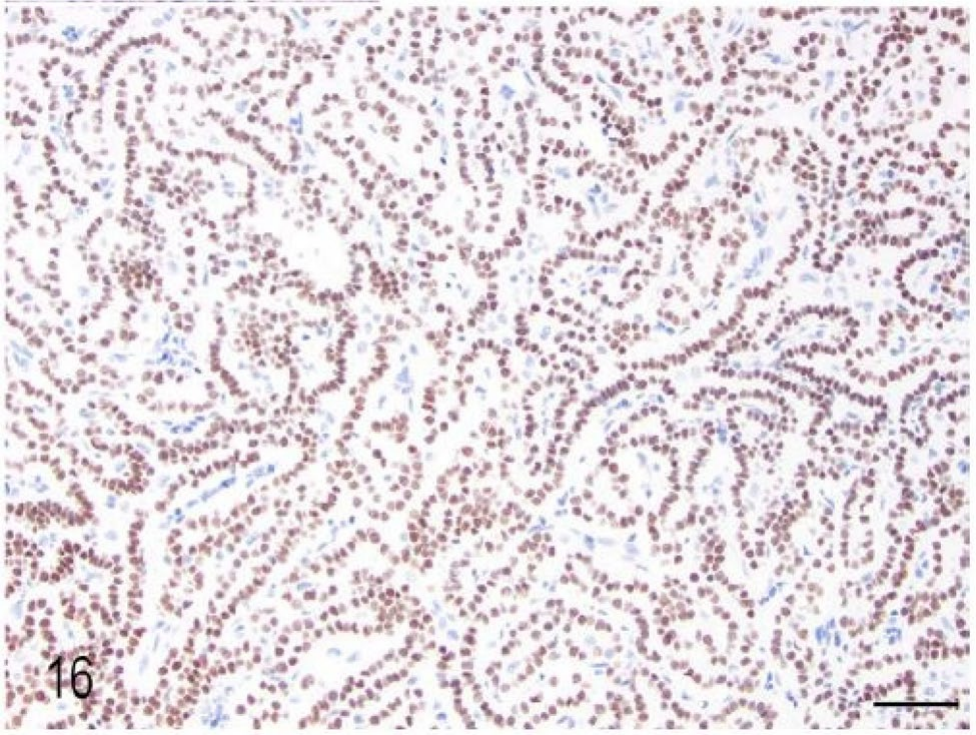
Case 3. The pulmonary adenoma was diffusely positive for TTF-1 immunohistochemistry. Bar = 50 µm.

Macroscopic pulmonary consolidation was observed in 4 animals (cases 2-4 and 8). Case 8 showed a focal, yellow, ill-defined nodule in the caudal right lung lobe (Figure 10). Case 2 showed unilateral, multifocal dark-reddening and consolidation of the right caudal lung lobe (Figure 11). Cases 3 and 4 showed multifocal and focal, respectively, well-demarcated, cream-white, firm nodules within the lung parenchyma (Figure 12). Microscopic examination of the lung from case 2 revealed pyogranulomatous and eosinophilic bronchointerstitial pneumonia with intra-bronchiolar and intra-alveolar nematode larvae that morphologically resembled *Angiostrongylus vasorum* (Figure 13). Nonetheless, qualitative PCR was negative. Histopathology of the lung tissue from cases 3 and 4 revealed multinodular or focal, well demarcated, non-encapsulated and expansile neoplastic proliferations diagnosed as bronchoalveolar adenomas (Figure 14). These neoplastic foci were immunohistochemically positive for MCK (Figure 15) and TTF-1 (Figure 16). EGFR was weakly positive. Scattered, strong nuclear positivity was observed for SOX-9 and Ki-67 (Ki-67 index of 0.5%). Neoplastic cells were negative for vimentin, PD1, PD-L1 and COX-2. Histopathology of the lung tissue from case 8 revealed a focal cholesterol granuloma. Case 5 exhibited a focally extensive pleural defect with fibrin exudation, suppurative pleuritis, multifocal hemorrhages and alveolar atelectasis.

## 4. Discussion

The present work summarises in detail the pathological findings of 8 captive meerkats (*Suricata suricatta)* that were submitted for PM examination to the RVC’s Diagnostic Laboratories over a period of 4 years. To the authors’ knowledge, this is the first comprehensive study that systematically analyses the causes of death in captive individuals of this species. Cases of unexpected death and euthanasia performed on welfare grounds were included and are in accordance with previous case reports described elsewhere (Bongiovann et al. 2016; Tomczyk and Zieliński 2021; Piewbang et al. 2022). Causes of sudden death that are reported in the literature are dilated cardiomyopathy due to taurine deficiency (Gutzmann et al. 2009), pancreatitis (Naples et al. 2010) or, less commonly, neoplasia (Singh et al. 2005). The cause of pancreatitis, however, remains to be fully elucidated but the nutritional status, namely daily energy intake and hyperlipidaemia, seem strong predisposing factors, particularly in captive animals. Interestingly, no evidence of pancreatitis was observed in any of the cases examined in the current study. Different types of neoplasia have been reported in captive meerkats, however, because only case reports have been published so far, their overall prevalence in this species remains unknown. Interestingly, 2 out of 8 cases (cases 3 and 4) showed multifocal or focal bronchoalveolar adenomas, diagnosis that was confirmed by the immunohistochemical profile, particularly the expression of TTF-1 and MCK. The negative or week expression of PD1, PD-L1, COX-2, EGFR and SOX-9 further support their benign nature. To the authors’ knowledge, this is the first immunohistochemical characterisation of pulmonary adenomas in meerkats. The pulmonary adenomas were considered in both cases as incidental findings and unrelated to the cause of death.

Two animals were in poor body condition when necropsied. Case 4 was an 8-yr-old female with a clinical history of neurological signs and the ante-mortem X-rays showed spondylosis. The necropsy of this individual revealed dental disease, which could account for the poor body condition due to prolonged anorexia. A precise cause for the clinically reported neurological signs could not be determined, however, pain associated with the spondylosis could potentially be attributed, as well as the worsening of the anorexia. Case 7 was a 2-yr-old male that was euthanised after presenting collapsed due to con-specific attack. Interestingly a trichophytobezoar within the stomach and an empty gastrointestinal tract were observed, and therefore anorexia due to ongoing bullying by another meerkat was considered most likely. Brox et al. described captive meerkats as clearly hierarchical and inherently competitive animals regarding food preferences within the group, which are, in turn, acquired through a pup-to-adult learning process. Inaccessibility of food leads to more uniform preferences and more likelihood for potential fighting between housed individuals (Brox et al. 2021). A similar clinical and pathological scenario was observed in another individual (case 6), in which skin abrasions and multifocal subcutaneous haematomas were grossly observed and a trichobezoar was found in the stomach. Additionally, this case presented with macroscopic and microscopic lesions of chronic dermatitis. Interestingly, the Toluidine Blue stain revealed increased numbers of mast cells in the dermis suggesting an underlying allergic dermatitis. It is noteworthy that 2 other animals in the present study (cases 1 and 8) showed plastic foreign bodies in the digestive tract leading to intestinal perforation and death and to euthanasia. These findings suggest that alimentary foreign bodies may not be uncommonly found in captive meerkats, especially gastric tricho(phyto)bezoars in cases of inter-specific attacks and bullying but also objects that may be accessible within the zoo enclosure. This data should raise concern amongst keepers not only to keep the zoo facilities free of foreign material susceptible to be ingested but also to provide the meerkats with appropriate environmental enrichment and enough food in order to avoid con-specific attacks.

Additionally, in the current work, spondylosis seemed to be a common incidental lesion in the aged meerkat, as supported by the literature (Sladky et al. 2000; Dadone et al. 2014) and could be potentially linked to clinical disease or otherwise remain subclinical.

Clinical neurological signs in meerkats with cholesterol granulomas are well described (Sladky et al. 2000; Piewbang et al. 2022). Herein we have described for the first time a case of systemic atherosclerosis with cerebral cholesterol granulomas and subsequent hydrocephalus (case 3). Gross findings included subcutaneous oedema, ascites and hydrothorax likely due to damaged vascular walls and increased vascular permeability. Histologically, auricular thrombosis and glomerulosclerosis were seen. Therefore, the cause of death in this animal was consistent with acute myocardial infarction due to ischaemia. The aetiopathogenesis of cholesterol granulomas is poorly understood except for the aged horse. However, as it seems to occur with pancreatitis, lipid-rich diets and hyperlipidaemia may play a role (Sladky et al. 2000) and, in this case, likely associated with systemic atherosclerosis. Studies focusing on the diet of meerkats in captivity could shed light on this condition and their possible association. Case 8 showed a focal pulmonary cholesterol granuloma, therefore, when they are either subclinical or unrelated to systemic atherosclerosis, they should be considered incidental findings.

Numerous infectious agents are reported to cause non-suppurative meningitis, encephalitis, or a combination of both lesions in captive and free-ranged meerkats. Viral causes include Japanese Encephalitis Virus and CDV (Coke et al. 2005; Piewbang et al. 2022) whilst protozoal encephalitis would include Toxoplasma (Basso et al. 2009). In the current study, an underlying demyelinating condition affecting the cerebral white matter, regardless of the specific cause, was ruled out by using the Luxol Fast Blue stain. In addition, one animal (case 2) that died unexpectedly showed histopathological lesions of lymphoplasmacytic meningitis and multifocal myocardial lymphocytic and neutrophilic infiltrates, but their precise aetiology could not be determined. This animal also showed a granulomatous verminous pneumonia that resembled that caused by *Angiostrongylus vasorum*. However, qualitative PCR targeting nematodes was negative, possibly due to poor DNA preservation in the formalin-fixed paraffin-embedded tissue block. Therefore, another possibility, although unproven, is that the myocardial infiltration was a result of an aberrant migration of the adult nematodes. Gillis-Germitsch et al. described that captive meerkat can be definitive hosts for *Angiostrongylus vasorum* and it is likely that they get infected after ingestion of the intermediate hosts (snails) that may be present within the zoo facilities. Similarly, systemic cestodiosis has been recently reported in a group of captive meerkats for the first time (McHale et al. 2020). This data ensures that facilities are kept as clean as possible as a matter of importance, with special emphasis on eliminating potential pests. Unlike canids, however, meerkats are usually subclinically infected with *Angiostrongylus vasorum* and are an unlikely cause of death in this species (Gillis-Germitsch et al. 2017). However, fatal disease has been attributed to muscular, peritoneal and pleural cestodiosis (genus *Spirometra* sp.) (McHale et al. 2020).

## 5. Conclusion

The present study emphasises non-infectious diseases like alimentary foreign bodies, con-specific attacks and systemic atherosclerosis, which is described for the first time, as causes of mortality in captive meerkats and highlights the importance of appropriate husbandry (e.g. environmental enrichment, cleaning of facilities and diet formulation) by zookeepers. Systemic inflammatory diseases suspected to be of an infectious origin are individually reported in the current study. Common incidental findings included pulmonary edema and congestion, cholesterol granulomas, pulmonary adenomas and vertebral spondylosis. This work warrants the need for larger studies of meerkat mortality in both captive and wild populations, of the behavioural and environmental factors that may contribute to population decline and highlights the importance of thorough pathological examinations, especially in cases of sudden death.
